# Pre-Medications for Non-Emergency Tracheal Intubation in the United States Neonatal Intensive Care Units

**DOI:** 10.7759/cureus.53512

**Published:** 2024-02-03

**Authors:** Mahmoud A Ali, Muppala Prasanth Raju, Greg Miller, Niraj Vora, Madhava Beeram, Venkata Raju, Ashith Shetty, Vinayak Govande, Nguyen Nguyen, Arpitha Chiruvolu

**Affiliations:** 1 Pediatrics/Neonatology, West Virginia University, Morgantown, USA; 2 Neonatology, Baylor Scott & White Health, Temple, USA; 3 Pediatrics, Baylor Scott & White Health, Temple, USA; 4 Neonatology, Baylor University Medical Center, Dallas, USA; 5 Neonatology, Pediatrix Medical Group, Dallas, USA

**Keywords:** fentanyl, procedure, policy, inherited neonate albinism, - elective, tracheal, pre-medication, mechanical ventilation, neonatal intensive care unit (nicu), pre-intubation

## Abstract

Background: Premedication in neonates undergoing elective intubation effectively minimizes the negative physiological events of bradycardia, systemic hypertension, intracranial hypertension, and hypoxia. Premedication decreases procedure-related pain and discomfort. This study aimed to evaluate the current practice of pre-intubation medications for non-emergent intubations in preterm and term neonates in the United States.

Study design: A cross-sectional survey (Appendix) was sent via e-mail to all level 3 and 4 Neonatal Intensive Care Units (NICUs) of the Organization of Neonatal Perinatal Medicine Training Program Directors (ONTPD), NICU directors with pediatric residency only, and Baylor Scott and White Health, Mednax, and Envision health services systems.

Results: Of 170 responses, 41% (69/168) routinely premedicate, 38% (64/168) premedicate under specific circumstances, and 21% (35/168) do not administer any routine pre-intubation medications. Only 46% (77/168) of units had a written policy. The most frequently used drugs were fentanyl (68%, 116/170), atropine (39%, 66/170), midazolam (38%, 64/170), and morphine (26%, 45/170). 21% (36/170) used a two-drug combination, and 38% (64/170) used a three-drug combination. The most commonly used two-drug combination was atropine and fentanyl, and the most common three-drug combination was atropine, fentanyl, and a paralytic agent.

Conclusion: Despite the well-documented benefits of premedication for NICU intubations, as aligned with AAP recommendations, the US lags behind other nations, with stagnant rates since 2006. This disparity persists despite a rise in written policies, which exhibit significant content variations. The authors advocate for the adoption of standardized, AAP-aligned policies across all NICUs in the US. Continued research is vital to monitor the progress of this crucial practice and address any underlying barriers to implementation.

## Introduction

Endotracheal intubation to resuscitate neonates was used by Scheel in 1798 [1]. Nowadays, endotracheal intubation is a universal procedure in neonatal intensive care units (NICUs). Intubation is a painful procedure [2], and it induces physiological stress, resulting in hypoxia, bradycardia, systemic and pulmonary hypertension, intracranial hypertension, and potential airway injury in neonates [3-9]. Repeated intubation attempts are of concern but happen frequently [10]. These risks are even more significant for preterm infants, with authors recently reporting an increased risk of severe intraventricular hemorrhage (IVH) and adverse neurodevelopmental outcomes in patients requiring multiple intubation attempts [11,12].

Previous studies have shown that premedication before non-emergent intubation in neonates reduces the adverse physiological responses, decreases the time and number of attempts needed to complete the intubation procedure, improves the intubation conditions, and minimizes the potential for intubation-related airway trauma [5,13-19]. However, despite the known benefits and the recommendations from major scientific societies such as the American Academy of Pediatrics (AAP), the Canadian Pediatric Society, and the International Evidence-Based Group for Neonatal Pain to premedicate infants before non-emergent intubation [5,16,20], there are wide variations in practice, frequency, and medications used before intubation [2]. In a survey from 2006 in the United States, only 44% of neonatology fellowship program directors reported routine use of analgesia and/or sedation before intubation [2]. In a more recent survey of members of the Perinatal Section of AAP, only 34% of neonatologists routinely premedicated before intubation [21].

The current study aims to evaluate the latest variations in premedication practice for non-emergent intubation in level 3 and 4 NICUs across the US, identifying factors associated with these variations and comparing them to previous practices.

## Materials and methods

This study included all level three and level four Neonatal Intensive Care Units (NICUs) in the United States. A ten-question survey (Attachment 1, Questions 1-10) was developed by the authors, submitted to the Organization of Neonatal Training Program Directors (ONTPD), and emailed to the 101 neonatology fellowship program directors. A modified survey with four more questions added (Attachment 1, Questions 1-14) was emailed to all the NICU medical directors of the 100 Accreditation Council for Graduate Medical Education (ACGME) accredited pediatrics residency training programs who do not have a neonatology fellowship program. The pediatric residency programs were identified via the National Resident Matching Program (NRMP) and the Association of American Medical Colleges (AAMC). The modified survey was also emailed to level III and level IV NICUs of Mednax, Envision, and Baylor Scott and White (BSW) Health. To sustain consistency and confirm that the respondents would be aware of the policies at their NICUs, only the fellowship program director or the NICU medical director of pediatrics residency programs were contacted. The original survey and two reminders were distributed via ONTPD in May and June 2020.

REDCap (Research Electronic Data Capture, v9.1.0) was used to collect the data with a link attached to the e-mail listings. SAS (version 9.4; SAS Institute, Cary, North Carolina) was used for all statistical analyses. Significance was indicated as p < 0.05. A chi-squared or Fisher’s exact test was used to test for associations in bivariate comparisons. A two-sample t-test, or Wilcoxon rank-sum test, was used to test for differences in continuous variables between two groups. The questionnaire and the study protocol were approved by the Institutional Review Board at Baylor Scott and White Health.

## Results

We obtained 170 responses. Of the 101 program directors contacted via ONTPD, 69 responded, for a response rate of 69%. Out of 100 pediatric residency programs, 34 responded with a response rate of 34%. Additionally, 67 NICUs from Mednax, Envision, and BSW Medical groups responded to the survey. Among the respondents, 41% (69/168) routinely premedicate all newborns before non-emergency intubation, while 21% did not practice any routine pre-medication, and 46% (77/168) had a written policy for preintubation medications. Out of the units that have a written policy, 65% (50/77) routinely premedicate all newborns before elective intubation versus 21% (19/91) of those who do not have a written policy. Out of units that have a written policy, 6% (5/77) admit to never using premedication, and 33% (30/91) of units that do not have a written policy admit to never using premedication (Table 1). 

**Table 1:** Differences in premedication regimens based on written policy N: number of responding NICUs. %: percentage of the responding NICUs. INSURE: Intubation, Surfactant, Extubation Chi square test is used for categorical variables

	Written policy N (%)	No written policy N (%)	p-value
Responding NICUs	77/168 (46%)	91/168 (54%)	
Routinely medicate all	50/77 (65%)	19/91 (21%)	
Not routinely medicate all	5/77 (6%)	30/91 (33%)	
Medicate for INSURE	2/77 (3%)	1/91 (1%)	
Using single drug	4/77 (5%)	19/91 (21%)	p=0.0032
Using two drugs	25/77 (32%)	11/91 (12%)	p=0.0014
Using three drugs	37/77 (48 %)	28/91 (31 %)	p=0.022
Fentanyl	64/77 (83%)	52/91 (57%)	p=0.0003
Atropine	51/77 (66%)	15/91 (16%)	p<0.0001
Midazolam	26/77 (34%)	38/91 (42%)	p=0.28
Morphine	15/77 (19%)	30/91 (33%)	p=0.049
Paralytic agent	50/77 (65%)	14/91 (15%)	p<0.0001
Time to prepare and give the medications			p=0.067
Less than 10 minutes	27/77 (36%)	36/71 (51%)	
10-15 minutes	27/77 (36%)	26/71 (37%)	
15-20 minutes	16/77 (21%)	4/71 (6%)	
20-30 minutes	5/77 (7%)	3/71 (4%)	
More than 30 minutes	1/77 (1%)	1/71 (1%)	
Side effects reported			p=0.32
No side effects 23% (34/149)	16/69 (23%)	18/80 (23%)	
side effects in less than 10% of cases 65% (97/149)	42/69 (69%)	55/80 (61%)	
side effects in more than 10% of cases 5% (7/149)	3/69 (4%)	4/80 (5%)	
Not sure about the incidence of side effects. 7% (11/149)	8/69 (12%)	3/80 (4%)	

Fentanyl was given in 83% of units that have a written policy versus 57% in units that do not (p-value=0.0003), atropine in 66% versus 16% (p-value<.0001), midazolam in 34% versus 42% (p-value =0.29), morphine in 19% versus 33% (p-value =0.049), and paralytic agents in 65% versus 15% (p-value<.0001) (Table 2). Table 3 shows drug combinations and side effects percentages.

**Table 2:** Premedication data from all survey responses ONTPD: Organization of Neonatal-Perinatal Medicine Program Directors. BSW: Baylor Scott and White health. N: number of responding NICUs. %: percentage of the responding NICUs. Chi-square test is used for categorical variables

Total respondents N (%)	170
ONTPD	69/170 (41%)
Residency NICU Director	34/170 (20%)
MEDNAX, Envision, BSW	67/170 (39%)
Routinely medicate all	69/168 (41%)
Not routinely medicate all	35/168 (21%)
Medicate for INSURE	3/168 (2%)
Atropine	66/170 (39%)
Morphine	45/170 (26%)
Midazolam	64/170 (38%)
Fentanyl	116/170 (68%)
Paralytics/Muscle Relaxants	64/170 (38%)
Lorazepam	5/170 (3%)
Propofol	4/170 (2%)
Pentobarbital	1/170 (0.6%)
Atropine given first in order	35/134 (27%)
Atropine given 2nd in order	21/111 (19%)
Atropine given 3rd in order	5/75 (7%)
Painkillers given first in order	72/134 (54%)
Painkillers given 2nd in order	55/111 (49%)
Painkillers given 3rd in order	8/75 (11%)
Sedation given first in order	23/134 (17%)
Sedation given 2nd in order	27/111 (24%)
Sedation given 3rd in order	11/75 (14%)
Paralytics/Muscle Relaxants first in order	0 (0 %)
Paralytics/Muscle Relaxants 2nd in order	10/111 (9%)
Paralytics/Muscle Relaxants 3rd in order	55/75 (72%)
two -drug combination	(36/170) 21 %
Atropine + Fentanyl	(12/170) 7 %
Midazolam + Fentanyl	(5/170) 3 %
Atropine + Morphine	(3/170) 2 %
Atropine + Paralytic agent	(3/170) 2 %
Atropine + Midazolam agent	(2/170) 1 %
Fentanyl + Paralytic agent	(2/170) 1 %
Midazolam + Morphine	(2/170) 1 %
Lorazepam + Morphine	(1/170) 0.6 %
Others	(6/170) 3 %
three -drug combination	(65/170) 40 %
Fentanyl + Atropine + Paralytic agent	(18/170) 10%
Fentanyl + Midazolam + Paralytic agent	(14/170) 8%
Morphine + Midazolam/lorazepam + Paralytic agent	(7/170) 4%
Fentanyl + Atropine + Midazolam/lorazepam	(4/170) 2%
Morphine + Atropine + Paralytic agent	(2/170) 1%
Morphine + Atropine + Midazolam	(1/170) 0.6 %
Morphine + Atropine + Pentobarbitol	(1/170) 0.6 %
Others	(18/170) 12 %
Time to prepare and give the medications	
Less than 10 minutes	63/146 (43%)
10-15 minutes	53/146 (37%)
15-20 minutes	20/146 (14%)
20-30 minutes	8/146 (5%)
More than 30 minutes	2/146 (1%)
When paralytic agents are not in use, the most common side effect reported was	
respiratory depression/apnea	(60%, 78/129)
chest wall rigidity	(26%, 34/129)
hypotension	(9%, 11/129).
no antidote to overcome side effects,	89% (127/143).
antidote needed to overcome side effects,	11% (16/143).
Muscle relaxants use if not part of guidelines	
None	50% (62/125)
< 10% of the elective procedures	35% (44/125)
> 10% of elective procedures	10% (13/125)
Academic institutions received the modified survey	(51%, 48/94).
Fellowship programs received the modified survey	(5%,5/94)
NICUs received the modified survey	
Level 3	63% (60/96)
Level 4	38% (36/96)
Geographic distribution	
South	49% (47/96)
West	20% (19/96)
Northeast	17% (16/96)
Midwest	15% (14/96)

**Table 3:** Drug combinations and side effects percentages N: number of the responding NICUs. %: percentage of the responding NICUs. Chi-square test used for categorical variables

incidence of side effects	2-drug combination (N (%)	3-drug combination N (%)
None	5/31 (16%)	19/54 (35%)
< 10% of cases	23/31 (74%)	30/54 (56%)
> 10% of cases	1/31 (39%)	0/54 (0%)
Not sure	2/31 (6%)	5/54 (9%)
P value	P=0.7	P=0.009

Among the NICUs that participated in our study, those with different fentanyl doses and infusion times did not show any significant differences in the rates of chest rigidity (Table 4).

**Table 4:** Chest rigidity in relation to Fentanyl dose and Fentanyl infusion time N: number of the responding NICUs. %: percentage of the responding NICUs. Mic/kg: microgram per kilogram. Chi-square test used for categorical variables

Fentanyl dose	N	% Chest rigidity	Fentanyl infusion	N	% Chest rigidity
0.5 mic/kg	9/126 (7%)	2/9 (22%)	2 Minutes	24/125 (19%)	3/24 (12%)
1 mic/kg	85/126 (67%)	17/85 (20%)	5 Minutes	64/125 (51%)	17/64 (27%)
2 mic/kg	28/126 (22%)	12/28 (43%)	10 Minutes	23/125 (18%)	7/23 (30%)
p-value		P=0.1			P=0.3

Out of the respondents, 23% (34/151) reported no side effects with the preintubation medications, 66% (99/151) reported side effects in less than 10% of cases, 5% (7/151) reported side effects in more than 10% of cases, and 7% (11/151) were not sure about the incidence of side effects. The difference in the incidence of adverse/side effects was not statistically significant for NICUs using a 2-drug combination (P=0.7) but was significant for those using a 3-drug combination (P=0.009). When paralytic agents are not used, the most common side effects reported were respiratory depression/apnea (60%, 78/129), followed by chest wall rigidity (26%, 34/129), and hypotension (9%, 11/129). 89% of NICUs reported no need for pharmacologic reversal to overcome these side effects.

Of the centers that are not using paralytic agents as part of their routine medications, 50% (62/125) did not use them at all, 35% (44/125) reported using them in less than 10% of the elective intubation procedures, and 10% (13/125) of those centers used them in more than 10% of elective procedures.

Premedication practices for newborns in US NICUs differ by region and level of care. Southern US NICUs is more likely to have a written policy on premedication than NICUs in the Northeast. Level 4 NICUs are more likely to routinely premedicate all newborns before elective intubations than level 3 NICUs (Table 5).

**Table 5:** Variation in preparation time of NICUs using one drug only, two drugs only and three drugs only regimen N: number of the responding NICUs. %: percentage of the responding NICUs. Chi-square test used for categorical variables

Difference in time needed to prepare in administer drugs/number of drugs used			
	One drug N (%)	Two drugs N (%)	Three drugs N (%)
< 10 minutes	13/63 (21%)	11/63 (17%)	26/63 (41%)
10-15 minutes	8/53 (15%)	15/53 (27%)	24/53 (45%)
15-20 minutes	1/20 (5%)	8/20 (40%)	9/20 (45%)
​​​​​20-30 minutes	1/8 (13%)	2/8 (25%)	4/8 (50%)
> 30 minutes	0/2 (0%)	0/2 (0%)	2/2 (100%)
p-value	p=0.5	p=0.25	p=0.58

## Discussion

Numerous studies have determined the benefits of premedication in neonates and that it should be considered a standard of care in non-emergency intubations, yet we report that the practice is not universal and there is considerable variation in the frequency and practice of premedication, yet the adverse effects of the premedication remain a clinician concern [22-24]. In 2019, a multicenter study conducted via the National Emergency Airway Registry for Neonates (NEAR4NEOS) showed that the tracheal intubation first attempt success rate in the NICU is 49%, site-specific tracheal intubation-associated event rates ranged from 9% to 50% (P < .001), and severe desaturation rates ranged from 29% to 69% (P =.001), despite advanced practice providers being the first airway providers for the majority of intubation procedures. Less adverse events were associated with paralytic premedication, video laryngoscope usage, and a higher training level of the providers [24].

Internationally, in Saudi Arabia, across the ten largest academic tertiary NICUs, even though 41% use premedication, only 26% have a written policy [22]. Although 70% believed it was essential to routinely use premedications for all elective intubations, 60% cited fear of potential side effects for avoiding premedication, and 40% indicated that the procedure could be executed more rapidly without drug therapy. Moreover, treatment regimens also varied widely among respondents [22]. In Malaysia, even though 97% of NICUs premedicate neonates for elective intubation, only 17% have a written policy [25]. While in the UK, the administration of pre-intubation medications was found to be routine in 100% of units in 2015, compared to 93% in 2007 and 37% in 1998. Also, 86% of units in the UK had preintubation guidelines in 2015, compared to 76% in 2007 and 14% in 1998 [26]. In the US, premedication usage was reported at 44% in 2006, 34% in 2015, and 41% in our survey. Also, written policies were adopted by 24%, 44%, and 46% in 2006, 2015, and 2020, respectively [2,22].

Our study is the most recent study evaluating the practice of premedication in the US, following two studies conducted in the last two decades by Sarkar (2006) and Muniraman (2015). Our study is different from the previous two studies on the target respondents. We surveyed program and medical directors to ascertain unit or program practices as a whole. 

We aimed to understand the current practice and experiences of level 3 and 4 NICUs regarding the use of premedication before non-emergent intubation. Our survey reveals the continued limited usage of preintubation medication. There was no improvement at all over almost two decades in both academic and non-academic institutions. In our report, 41% of units routinely premedicate newborns before elective intubation, compared to 44% reported by Sarkar in 2006 and 34% reported by Muniraman in 2015. We also report an increasing number of units implementing a written policy, 46% in our report compared to 24% in 2006 and 44% in 2015 (2). In our report, the use of premedication was significantly higher in units with a written policy (65%) than those who do not (21%). 72% of those who routinely premedicate before elective intubation admitted having a written policy for that, compared to 47% reported by Sarkar, which highlights the significance of having a written policy on premedication use (p value<0.001). Out of level 4 NICUs, 49% routinely premedicate all newborns before elective intubations, compared to 37% of level 3 NICUs (p value = 0.28), which did not increase since Sarkar`s study in 2006 and is way less than the UK report of 2015.

We demonstrated the persistent variation in the premedication regimen. The most commonly used drug is fentanyl (68%), and it was the most commonly used drug reported by Muniraman in 2015 (79%). The high use of fentanyl might be explained by its rapid onset of action and the co-administration of paralytic agents masking the potential chest wall/laryngeal rigidity (14, 29). The AAP 2010 statement described the use of fentanyl as preferred, compared to morphine or remifentanil [16]. It is interesting that our report did not indicate any statistical difference in the incidence of side effects, regardless of the given dose of fentanyl or the duration of the infusion. Atropine use increased to 39% in our study compared to 25% in the US in 2015, whereas its use was 67% in the UK in 2015 (26). The increased usage of atropine may reflect its protective effect against bradyarrhythmia secondary to vagal stimulation (16, 30).

Paralytic agent usage slightly increased to 38%, up from 25% in 2006 and 34% in 2015, while in the UK, 92% (122/132 units) included a paralytic agent in their usual regimen in 2015. Although paralytic agents can precipitate respiratory muscle paralysis and render positive pressure ventilation challenging, the strikingly high use of paralytic agents in the UK and the increased use in the US could be due to the benefit in the situation of possible laryngospasm or chest wall rigidity associated with fentanyl administration, especially if they are given in combination. Also, there is evidence that paralytic agent use is associated with decreased moderate and severe tracheal intubation-adverse events in neonates [27].

Inadequate preparation and administration time have been recognized as potential reasons for not using premedication, but in a non-emergent situation, adequate ventilation can be provided while drugs with a relatively quick onset of action can be administered. In our study, 36% of NICUs who have a written policy spent less than 10 minutes preparing and administering the medications, compared to 51% of those who do not have a written policy. This is possibly related to providers` choice in units that do not have a written policy to use readily accessible medications, while those practicing in units with a written policy probably prefer the medications in the policy even if it would take a longer time. We assume that the time required will vary based on the type of medication(s) used, the dose and duration of the infusion, and availability in the NICU versus delivery from the pharmacy. Single drug versus multiple drugs would seem likely to alter the time required, although the number of drugs used did not affect the duration of administration (p value=0.5).

Premedication for INSURE is recommended to avoid intubation-related adverse events. Clinicians who have concerns about failed extubation because of the premedication effects might consider using drugs with rapid onset and a shorter duration of action [23,28].

Our study has an ample number of respondents to be representative of the use of preintubation medications in the NICUs in the US. Possible limitations to this study include missing data as not all NICUs answered all the questions of the survey and the demographic questions were added to the modified survey; the possibility of duplicated responses; a lack of compliance with the written policy; and limiting the survey to levels 3 and 4. Not all NICUs answered all the questions of the survey, which led to several missed data points. However, there was no particular pattern in the missing data. The possibility of duplicate responses, although unlikely, is still possible, as there are a considerable number of NICUs at ACGME-accredited residency and/or fellowship programs also affiliated with Mednax or Envision medical groups. To avoid duplicate responses, the respondents were instructed not to do the survey if they had already completed it via another forum. Although the medical directors of the NICU are aware of their unit’s policy, other providers, including neonatologists, nurse practitioners, respiratory therapists, and fellows, may choose not to follow the policy for various reasons, which may cause actual practice to differ from the perception of the medical director. Limiting the survey to level 3 and 4 units may give an incomplete picture of the use of premedications in the US in view of the large number of level 2 NICUs and transport teams that electively intubate babies before referring them to higher-level institutions. However, targeting level 3 and 4 NICUs, where most of the non-emergent intubations take place, and targeting only one person in each unit gave a significant strength to our survey. Furthermore, for unknown reasons, 126 respondents reported fentanyl dosage, while only 116 reported using fentanyl in pre-intubation medication.

Having a written policy is one of the most influential factors in improving the practice of premedication for elective intubation. NICUs with a written policy are far more likely to routinely premedicate before elective intubation.

## Conclusions

Despite the awareness of the AAP recommendation for preintubation medications and the acknowledgment of their benefits, routine use of preintubation medications in the US has not improved since 2006. Preintubation medication practice in the US is less prevalent than in Western European countries. The adoption of written policy is increasing and is associated with a greater likelihood to premedicate. There is a wide variation in the premedication regimens. To ensure adherence to the AAP's recommendations, the authors urge all NICUs to formally establish a written policy for using preintubation medications. Implementing additional strategies like monitoring, training, and quality control measures is crucial for optimal implementation. Future studies with larger response rates, including level 1 and 2 NICUs and transport teams, will help to monitor this practice in the US.

## Appendices

**Table 6:** 14-questions survey distributed during our study

Original survey includes questions 1-10, Modified survey include questions (1-14)
Question 1: Do you routinely pre-medicate newborns before ELECTIVE intubation?
Yes, to all intubation
No to all
Yes for INSURE (Intubate -Surfactant Extubate)
Yes, for specific conditions or anticipated difficult intubation
Yes, for specific age groups. Please specify the age groups:
Question 2: Do you have a written policy or guidelines for pre-initiation medications?
Yes
No
Question 3: What is/are the medication(s) you are using? (Please check all that applies)
Atropine
Morphine
Midazolam
Fentanyl
Paralytic agents/skeletal muscle relaxants
Other, please specify;
Question 4: What is the time required for the medications to be prepared and administered before intubation?
Less than 10 minutes
10 - 15 minutes
15 - 20 minutes
20 - 30 minutes
More than 30 minutes
Question 5: What is the order of Medications administration?
First drug given is: --------------------------
Second drug given is: -----------------------
Third drug given is: -------------------------
Fourth drug given is (if applicable): ------------
Question 6: If You are using fentanyl as one of your pre-intubation medications (Please answer if applicable)
6A: The used fentanyl dose is:
0.5 mic/kg
1 mic/kg
2 mic/kg
Other; Please specify
6B: Fentanyl dose is infused over
2 minutes
5 minutes
10 minutes
Other; Please specify
6C: Other, please specify:
Question 7: In your experience, what is your estimated percentage of infants with side effects due to these medications?
None or 0%
Less than 10%
More than 10%
Not sure
Question 8: In your experience, what is the most frequent side effect encountered?
Respiratory depression/Apnea (Please choose this answer only if paralytic agents/muscle relaxants is not part of your routine pre-intubation medications?
Chest wall rigidity
Laryngospasm
Hypotension
Other (please specify)
Question 9: Did you need to give an antidote to reverse this/these side effects
No
Yes, Please Specify:
Question 10: If not part of your pre-intubation medications, how % often do you use skeletal muscle relaxants or paralytic agents before elective intubation?
None or 0%
Less than 10%
More than 10%
Only in specific conditions (Please, Specify)
Question 11: Is your institution a teaching hospital (medical students and/ or residents/fellows)?
Yes
No
Question 12: Does your practice have a neonatal fellowship program?
Yes
No
Question 13: What is the NICU level where you practice?
I
II
III
IV
Question 14: What region of the United States is your NICU t located?
Northeast
West
South
Midwest
